# Supplementary material for: Amino acid sequence assignment from single molecule peptide sequencing data using a two-stage classifier
Source: PLoS Comput Biol. 2023 May 30;19(5):e1011157. doi: 10.1371/journal.pcbi.1011157 (PMC10256185; doi:10.1371/journal.pcbi.1011157)
Supplement: S1 Table — (PDF) [file pcbi.1011157.s001.pdf]

$\boldsymbol{f}^{(t)}$	Cumulative probabilities for each state at timestep t
$\boldsymbol{\mathcal{f}}^{(t)}$	Tensor form of $\boldsymbol{f}^{(t)}$
$\boldsymbol{O}^{(t)}$	HMM Emission matrix for timestep t
$\boldsymbol{\mathcal{O}}^{(t)}$	Tensor form of $\boldsymbol{O}^{(t)}$
$\widehat{\boldsymbol{\mathcal{O}}}^{(t)}$	A pruned approximation of $\boldsymbol{\mathcal{O}}^{(t)}$
$\boldsymbol{T}$	The HMM transition matrix
$\boldsymbol{\mathcal{T}}$	Tensor form of $\boldsymbol{T}$
$\widehat{\boldsymbol{\mathcal{T}}}^{(t)}$	A pruned approximation of $\boldsymbol{\mathcal{T}}$ at timestep t
T	The number of timesteps
$Y_{1:T}$	Random variables representing the series of observations
$y_{1:T}$	The true values of $Y_{1:T}$
$X_{1:T}$	Random variables representing the state of the HMM across time
Z	Random variable representing the peptide
z	True value of Z
s	The number of states of a fluorophore
Λ	The number of fluorophores
C	The number of colors of fluorophore
Λ_c	The number of fluorophores of color c
ρ	Number of amino acids successfully removed by Edman degradation
$\bar{c}$	Color of N-terminal amino acid
$\lambda_{\rho,c}$	Number of amino acids which can accept a label of color c when ρ amino acids have been removed from the peptide
ϕ_c	Number of functioning fluorophores of color c remaining for the peptide
α	Number of amino acids in the peptide (before sequencing)
p_c	Fluorophore loss rate for color c
$\boldsymbol{\mathcal{B}}^{(c)}$	A factored component of $\boldsymbol{\mathcal{T}}$ representing loss of fluorophores of color c
$\widehat{\boldsymbol{\mathcal{B}}}^{(t,c)}$	A pruned approximation of $\widehat{\boldsymbol{\mathcal{B}}}^{(c)}$ at timestep t
e	Edman cycle failure rate
$\boldsymbol{\mathcal{E}}$	A factored component of $\boldsymbol{\mathcal{T}}$ representing Edman degradation success and failure
$\widehat{\boldsymbol{\mathcal{E}}}^{(t)}$	A pruned approximation of $\boldsymbol{\mathcal{E}}$ at timestep t
d	Peptide detachment rate
$\boldsymbol{\mathcal{D}}$	A factored component of $\boldsymbol{\mathcal{T}}$ representing peptide detachment
$\widehat{\boldsymbol{\mathcal{D}}}^{(t)}$	A pruned approximation of $\boldsymbol{\mathcal{D}}$ at timestep t
r	Number of values of $\boldsymbol{\mathcal{O}}$ kept during pruning
r_c	Number of fluorophore counts of color c kept during pruning
$r_c^{(t)}$	r_c at time t
$\bar{r}$	Number of amino acid counts kept during pruning
$\bar{r}^{(t)}$	$\bar{r}$ at time t
h	The number of peptides selected by the kNN in the hybrid model
ζ_h	The set of peptides selected by the kNN in the hybrid model
