# Supplementary material for: Amino acid sequence assignment from single molecule peptide sequencing data using a two-stage classifier
Source: PLoS Comput Biol. 2023 May 30;19(5):e1011157. doi: 10.1371/journal.pcbi.1011157 (PMC10256185; doi:10.1371/journal.pcbi.1011157)
Supplement: S1 Appendix — (PDF) [file pcbi.1011157.s002.pdf]

HMM state space reduction

The basics

For fluorophores that are distinguishable and dependent on each other, where s is the number of states of one fluorophore, and Λ is the number of fluorophores, the number of states modeled in the system is given as in [1] by:

$$s^{\Lambda} \tag{7}$$

In contrast, if the fluorophores are indistinguishable and independent of each other, the number of states modeled in the system is instead given by the combinatoric equation from [1]:

$$\binom{\Lambda + s - 1}{s - 1} \tag{8}$$

Imaging is typically performed with high concentrations of the antioxidant Trolox [2] and for relatively short time intervals (100 msec); reversibly photobleached fluorophores do not occur frequently in our data and we ignore them as a first approximation. Therefore, we take s to be 2, with one state for a functioning fluorophore, and another for a missing, photobleached, or chemically destroyed fluorophore. This reduces (8) to $\Lambda + 1$ states given Λ indistinguishable and independent fluorophores.

More colors

Obviously, a red fluorophore is distinguishable from a blue one. But we would still like to benefit from the indistinguishability of red fluorophores from red fluorophores, and of blue fluorophores from blue fluorophores. This is modeled by first considering the states for each color of fluorophore independently, and then taking the cartesian product of these state spaces. For C colors of fluorophore, where Λ_c is the number of fluorophores of color c, this results in the number of states given by:

$$\prod_{c=1}^{C}(\Lambda_c + 1) \tag{9}$$

Each state then represents the number of remaining active fluorophores for each of our C colors of fluorophore.

Edman degradation

Our second challenge is the inclusion of Edman degradation in the HMM. Sequential removal of the N-terminal amino acid from each peptide breaks the assumption of indistinguishable fluorophores, which is the basis for the state reduction performed in [12]. However, through inductive reasoning we show that our model meets a weaker criterion, which can be used to merge physical states together as desired:

Any two physical states with the same numbers of fluorophores of each color and the same number of amino acids are equally likely. (10)

If we ignore Edman degradation, this follows directly from the assumed indistinguishability property of fluorophores of the same color; if two fluorophores behave identically, they are equally likely to be missing, photobleached, or chemically destroyed, thus it follows by symmetry that any two physical states with the same numbers of indistinguishable fluorophores of each color are equally likely. If we consider Edman degradation, then (10) is true for all states where no amino acids have yet been successfully removed. Let ρ indicate the number of amino acids removed from the original peptide. We have then shown that (10) is true when $\rho = 0$.

If physical states with identical fluorophore counts are equally probable for all states with ρ amino acids removed, it can be shown that all physical states with equal fluorophore counts are equally probable for all states with $\rho + 1$ amino acids removed. For removal of an amino acid that can't accept fluorophores

under the experimental setup this is trivial, so consider a peptide with ρ removed amino acids, an N-terminal amino acid which accepts fluorophores of color $\bar{c}$, and $\lambda_{\rho,\bar{c}}$ amino acids total which can accept a label of color $\bar{c}$. Then let $\phi_{\bar{c}}$ represent the number of remaining functional fluorophores for the peptide, satisfying $0 \leq \phi_{\bar{c}} \leq \lambda_{\rho,\bar{c}}$.

There are several conditions of the peptide with $\phi_{\bar{c}}$ functioning fluorophores scattered among the $\lambda_{\rho,\bar{c}}$ amino acids that can accept a label. When we remove the N-terminal amino acid, we may or may not remove with it a functioning fluorophore. The physical states which do have a functioning fluorophore in the N-terminal position (only possible when $\phi_{\bar{c}} > 0$) will have their other $\phi_{\bar{c}} - 1$ fluorophores distributed between the $\lambda_{\rho,\bar{c}} - 1$ remaining amino acids which can be labeled. Furthermore, these physical states are equally likely, as they are a subset of the equally likely physical states with $\phi_{\bar{c}}$ fluorophores. Since trivially $\lambda_{\rho,\bar{c}} - 1 = \lambda_{\rho-1,\bar{c}}$, these physical states map one-to-one with the physical states for the peptide with one less amino acid remaining, when it has $\phi_{\bar{c}} - 1$ dyes.

When $\phi_{\bar{c}} < \lambda_{\rho,\bar{c}}$, there are physical states with no fluorophore in the N-terminal position, even though the N-terminal amino acid can accept one. Then the $\phi_{\bar{c}}$ fluorophores will be distributed with equal probabilities among the $\lambda_{\rho,\bar{c}} - 1 = \lambda_{\rho-1,\bar{c}}$ remaining amino acids which can be labeled. Similarly to the other case, these physical states map one-to-one with the physical states for the peptide less one amino acid when it has $\phi_{\bar{c}}$ dyes.

The equally distributed probabilities and one-to-one correspondence between physical states across this amino acid removal ensures that these transformations do not break our guarantees of equal probabilities for $\rho + 1$ amino acids removed. Iteratively applying this reasoning, starting with $\rho = 0$, until we prove that physical states where $\rho = \alpha$ are equally likely if they have the same fluorophore counts, demonstrates that (5) is true under the assumptions we have taken.

This proves (10), which allowed us to safely reduce physical states that share both the same fluorophore counts by color and the same numbers of amino acids into a single modeled state.

Transition probabilities

We also need to know the transition probabilities for our new reduced state space. To deal with peptide detachment is trivial. Dye-loss, either for dyes missing before sequencing begins, or from chemical destruction during sequencing, can be modeled with a binomial distribution. This follows from the assumption that the fluorophores behave independently of each other.

For Edman degradation, there is of course a probability of success or failure of the degradation step, which we model as a Bernoulli random variable. In the case of success, we employ an additional Bernoulli random variable to model the probability of losing or not losing a functioning fluorophore. Because the physical states within a modeled state are equally likely, we can use combinatorics to count the number of states which will lose a dye, and the number that won't. Together these values can be used to find the probability of losing a fluorophore given a successful Edman degradation, as shown in the following formula, which conveniently reduces to a simple fraction:

$$\frac{\binom{\lambda_{\rho,\bar{c}}-1}{\phi_{\bar{c}}-1}}{\binom{\lambda_{\rho,\bar{c}}}{\phi_{\bar{c}}}} = \frac{\phi_{\bar{c}}}{\lambda_{\rho,\bar{c}}} \tag{11}$$

State reduction conclusions

This state reduction provides a considerable algorithmic complexity improvement to the HMM forward algorithm. The complexity of the forward algorithm is $O(S^2T)$, where S is the number of states, and T is the number of timesteps. Then, if implemented with the physical state space of a labeled peptide, the number of states S is $O(\alpha 2^{\Lambda})$, and we get a complexity of $O(\alpha^2 4^{\Lambda} T)$ for the HMM forward algorithm, where α is the number of amino acids and Λ is the total number of fluorophores (of any color).

However, if we reduce to our modeled state space, then S is $O\left(\alpha \prod_{c=1}^{C} \Lambda_c\right)$, giving an algorithmic complexity of $O(\alpha^2 (\prod_{c=1}^{C} \Lambda_c^2) T)$ for the forward algorithm, where C is the number of fluorophore colors being used and Λ_c is the number of fluorophores of color c. The scaling in either case is dominated by values of Λ or Λ_c, which ranges from 1 to about 25 for human tryptic peptides, though in rare cases Λ_c can exceed 100.

References

1. Messina TC, Kim H, Giurleo JT, Talaga DS. Hidden Markov Model analysis of multichromophore photobleaching. *The Journal of Physical Chemistry B*. 2006;110(33):16366-16376.
2. Swaminathan J, Boulgakov A, Hernandez ET, Bardo AM, Bachman JL, Marotta J, *et al.* Highly parallel single-molecule identification of proteins in zeptomole-scale mixtures. *Nature Biotechnology*. 2018;36(11):1076-1082.
