# Supplementary material for: Amino acid sequence assignment from single molecule peptide sequencing data using a two-stage classifier
Source: PLoS Comput Biol. 2023 May 30;19(5):e1011157. doi: 10.1371/journal.pcbi.1011157 (PMC10256185; doi:10.1371/journal.pcbi.1011157)
Supplement: S2 Appendix — (PDF) [file pcbi.1011157.s003.pdf]

Transition matrix factoring

The concept

Multiplication by sparse matrices is far more efficient than with dense matrices. Matrix vector multiplication with a dense matrix is $O(S^2)$ where S is the size of the vector; for this application vectors with thousands of entries are not uncommon, and even larger vectors are possible, although this depends on the protease and labeling scheme used. For a sparse matrix, matrix vector multiplication can be made to be $O(V)$, where V is the number of non-zero entries in the matrix. For highly sparse matrices this can be a significant improvement.

Since peptides cannot gain amino acids or functioning fluorophores during sequencing, a basic transition matrix for fluorosequencing has zeros except for entries for transitions in which the numbers of fluorophores of each color is decreasing or staying the same. While this does reduce the number of necessary operations, it only does this by a constant factor, with no effect on the asymptotic behavior in the limit. Additionally, the number of amino acids either stays the same, decreases by one (from a successful Edman degradation), or decreases to zero (from a peptide detachment event). This did improve the asymptotic behavior in the number of non-zero entries of the transition matrix, reducing this from $O\left(\alpha^2 \prod_{c=1}^{C} \Lambda_c^2\right)$ to $O\left(\alpha \prod_{c=1}^{C} \Lambda_c^2\right)$.

However, we did better by factoring this matrix (Fig 4). We used the independence of our different forms of error, with one matrix in the factored product for each type of error. To demonstrate this factorization, we reformulated our problem in tensor notation. The vector for the state space of a peptide with C colors not undergoing Edman degradation or peptide detachment can be viewed as a tensor of order C. Each index of the tensor maps to the fluorophore counts of a different color, and the value of an index i_c indicates the number of functioning fluorophores of color c, and satisfies $0 \leq i_c \leq \Lambda_c$. We also have indices j_c which are similarly defined. Since the transition matrix is a linear mapping

from and to this tensor of order C, it is necessarily of order $2C$. We use the Einstein summation convention, and three multi-indices $\boldsymbol{i} = i_1 i_2 \dots i_C$ and $\boldsymbol{j} = j_1 j_2 \dots j_C$ and $\boldsymbol{k} = k_1 k_2 \dots k_C$ for convenience. The matrix-vector multiplication operation for one step of the HMM forward algorithm is then given by:

$$\boldsymbol{\mathcal{f}}_{\boldsymbol{k}}^{(t+1)} = \boldsymbol{\mathcal{O}}_{\boldsymbol{kj}}^{(t+1)} \boldsymbol{\mathcal{T}}_{\boldsymbol{ji}} \boldsymbol{\mathcal{f}}_{\boldsymbol{i}}^{(t)} \tag{12}$$

Where (t) and $(t+1)$ indicate the timestamp of the values in the order C tensor $\boldsymbol{\mathcal{f}}^{(t)}$, which is indexed by the numbers of working fluorophores for each color and is the tensor form of $\boldsymbol{f}$ from (1), $\boldsymbol{\mathcal{T}}$ is the transition matrix $\boldsymbol{T}$ converted into tensor form, $\boldsymbol{\mathcal{O}}$ is the emission matrix $\boldsymbol{O}$ converted into tensor form.

Considering fluorophore loss only

Assuming no interactions between different fluorophores and ignoring Edman degradation and peptide detachment, $\boldsymbol{\mathcal{T}}$ satisfies the following equation:

$$\boldsymbol{\mathcal{T}}_{\boldsymbol{ji}} = \begin{cases} \prod_{c=1}^{C} \binom{i_c}{j_c} p_c^{i_c - j_c} (1 - p_c)^{j_c}, & \text{if } \boldsymbol{j} \le \boldsymbol{i} \\ 0, & \text{otherwise} \end{cases} \tag{13}$$

Where p_c is the per cycle dye loss rate of the fluorophores for color c. This is simply the product of the binomial distributions for each indexed color of fluorophore. To improve the sparsity of this representation, we can factor $\boldsymbol{\mathcal{T}}$ into second order tensors $\boldsymbol{\mathcal{B}}^{(1)} \boldsymbol{\mathcal{B}}^{(2)} \dots \boldsymbol{\mathcal{B}}^{(C)}$ such that:

$$\boldsymbol{\mathcal{B}}_{ji}^{(c)} = \begin{cases} \binom{i}{j} p_c^{i-j} (1 - p_c)^{j}, & \text{if } j \le i \\ 0, & \text{otherwise} \end{cases} \tag{14}$$

This produces a factorization of $\boldsymbol{\mathcal{T}}$:

$$\boldsymbol{\mathcal{T}}_{\boldsymbol{ji}} = \boldsymbol{\mathcal{B}}_{j_1 i_1}^{(1)} \boldsymbol{\mathcal{B}}_{j_2 i_2}^{(2)} \dots \boldsymbol{\mathcal{B}}_{j_C i_C}^{(C)} \tag{15}$$

We can plug this into (12) and find:

$$\boldsymbol{f}_{\boldsymbol{j}}^{(t+1)} = \boldsymbol{\mathcal{B}}_{j_1 i_1}^{(1)} \boldsymbol{\mathcal{B}}_{j_2 i_2}^{(2)} \ldots \boldsymbol{\mathcal{B}}_{j_C i_C}^{(C)} \boldsymbol{f}_{\boldsymbol{i}}^{(t)} \tag{16}$$

This reduces the algorithmic complexity in this simple case from $O\left(\prod_{c=1}^{C} \Lambda_c^2\right)$ to $O\left(\left(\prod_{c=1}^{C} \Lambda_c\right)\left(\sum_{c=1}^{C} \Lambda_c\right)\right)$.

Fluorophore loss and Edman degradation

We can expand on this to consider the Edman degradation: In that case we need more indices for the number of remaining amino acids. We modify (12) with additional indices u and v which satisfy $0 \leq u \leq \alpha$ and $0 \leq v \leq \alpha$, indicating the number of successful amino acid removals, or alternatively the position of an amino acid in the peptide (i. e., the amino acid at the N-terminus of the peptide when u amino acids have been removed). This gives:

$$\boldsymbol{f}_{\boldsymbol{vk}}^{(t+1)} = \boldsymbol{\mathcal{O}}_{\boldsymbol{kj}}^{(t+1)} \boldsymbol{\mathcal{T}}_{\boldsymbol{vjui}} \boldsymbol{f}_{\boldsymbol{ui}}^{(t)} \tag{17}$$

Note that the emission tensor $\boldsymbol{\mathcal{O}}$ is unaffected by the amino acid count, and depends only on the fluorophore counts, so it does not need to be modified.

We modify $\boldsymbol{\mathcal{T}}$ from (13) to model Edman degradation, and the exact form of $\boldsymbol{\mathcal{T}}$ will depend on the peptide under consideration. Let $\bar{c}_u$ be a number indicating the color of the fluorophore at position u in the peptide, with a value of 0 indicating no fluorophore, and let $\lambda_{u,\bar{c}_u}$ indicate the number of fluorophores of color $\bar{c}_u$ remaining when $u-1$ amino acids have been removed from the peptide. Then $\boldsymbol{\mathcal{T}}$ is defined by:

$$\boldsymbol{\mathcal{T}}_{\boldsymbol{vjui}} = \begin{cases} e\beta(\boldsymbol{i},\boldsymbol{j}), & \text{if } \boldsymbol{j} \leq \boldsymbol{i} \text{ and } v = u \\ (1-e)\beta(\boldsymbol{i},\boldsymbol{j}), & \text{if } \boldsymbol{j} \leq \boldsymbol{i} \text{ and } v = u+1 \text{ and } \bar{c}_u = 0 \\ (1-e)\left(\left(1 - \dfrac{i_{\bar{c}_u}}{\lambda_{u,\bar{c}_u}}\right)\beta(\boldsymbol{i},\boldsymbol{j}) + \left(\dfrac{i_{\bar{c}_u}}{\lambda_{u,\bar{c}_u}}\right)\bar{\beta}(\boldsymbol{i},\boldsymbol{j},u)\right), & \text{if } \boldsymbol{j} \leq \boldsymbol{i} \text{ and } v = u+1 \text{ and } \bar{c}_u > 0 \\ 0, & \text{otherwise} \end{cases} \tag{18}$$

Where:

$$\beta(\boldsymbol{i},\boldsymbol{j}) = \prod_{c=1}^{C} \binom{i_c}{j_c} p_c^{i_c - j_c} (1 - p_c)^{j_c} \tag{19}$$

And:

$$\bar{\beta}(\boldsymbol{i},\boldsymbol{j},u) = \binom{i_{\bar{c}_u} - 1}{j_{\bar{c}_u}} p_{\bar{c}_u}^{i_{\bar{c}_u} - 1 - j_{\bar{c}_u}} \left(1 - p_{\bar{c}_u}\right)^{j_{\bar{c}_u}} \prod_{\substack{1 \le c \le C \\ c \ne \bar{c}_u}} \binom{i_c}{j_c} p_c^{i_c - j_c} (1 - p_c)^{j_c} \tag{20}$$

The probability of an Edman degradation failure is essentially the same as in (13), but multiplied by e to account for the probability of failure. The probability for a transition involving a successful Edman degradation event which removes an unlabelable amino acid is similarly just like in (13) but multiplied by $(1 - e)$, the probability of success. If the amino acid in question is labelable by a color $\bar{c}_u$, then we may or may not remove a fluorophore of that color in the transition, so we need to take the sum of both possibilities. β in (19) gives the standard product of binomials formula from (13), but needs to be multiplied by the probability of no dye loss, which in (18) is $\left(1 - \frac{i_{\bar{c}_u}}{\lambda_{u,\bar{c}_u}}\right)$. This is then summed with $\bar{\beta}$ from (20) which gives the product of binomial probabilities starting with one less fluorophore of the color $\bar{c}_u$, which in (18) is multiplied with the probability of losing a fluorophore with the Edman degradation, $\frac{i_{\bar{c}_u}}{\lambda_{u,\bar{c}_u}}$. The sum of these two possibilities is then multiplied by the probability of an Edman degradation success, given by $(1 - e)$.

To make this more efficient, we introduce a new tensor $\boldsymbol{\mathcal{E}}$ which represents a transformation for Edman degradation. We define tensor $\boldsymbol{\mathcal{E}}$ as:

$$\boldsymbol{\mathcal{E}}_{v\boldsymbol{k}u\boldsymbol{j}} = \begin{cases} e, & \text{if } v = u \text{ and } \boldsymbol{k} = \boldsymbol{j} \\ 1 - e, & \text{if } v = u + 1 \text{ and } \boldsymbol{k} = \boldsymbol{j} \text{ and } \bar{c}_u = 0 \\ (1 - e)\left(1 - \dfrac{j_{\bar{c}_u}}{\lambda_{u,\bar{c}_u}}\right), & \text{if } v = u + 1 \text{ and } \boldsymbol{k} = \boldsymbol{j} \text{ and } \bar{c}_u > 0 \\ (1 - e)\left(\dfrac{j_{\bar{c}_u}}{\lambda_{u,\bar{c}_u}}\right), & \text{if } v = u + 1 \text{ and } k_{\bar{c}_u} = j_{\bar{c}_u} - 1 \text{ and } k_c = j_c \forall c \ne \bar{c}_u \text{ and } \bar{c}_u > 0 \\ 0, & \text{otherwise} \end{cases} \tag{21}$$

This provides the following factorization of $\boldsymbol{\mathcal{T}}$:

$$\boldsymbol{\mathcal{T}}_{\boldsymbol{vkui}} = \boldsymbol{\mathcal{E}}_{\boldsymbol{vkuj}} \boldsymbol{\mathcal{B}}^{(1)}_{j_1 i_1} \boldsymbol{\mathcal{B}}^{(2)}_{j_2 i_2} \dots \boldsymbol{\mathcal{B}}^{(C)}_{j_C i_C} \tag{22}$$

By substituting into (17) and adding an additional multi-index $\boldsymbol{l} = l_1 l_2 \dots l_C$ we get:

$$\boldsymbol{f}^{(t+1)}_{\boldsymbol{vl}} = \boldsymbol{\mathcal{O}}^{(t+1)}_{\boldsymbol{lk}} \boldsymbol{\mathcal{E}}_{\boldsymbol{vkuj}} \boldsymbol{\mathcal{B}}^{(1)}_{j_1 i_1} \boldsymbol{\mathcal{B}}^{(2)}_{j_2 i_2} \dots \boldsymbol{\mathcal{B}}^{(C)}_{j_C i_C} \boldsymbol{f}^{(t)}_{\boldsymbol{ui}} \tag{23}$$

Despite its high dimensionality, $\boldsymbol{\mathcal{E}}$ is highly sparse, with no more than three non-zero entries per column (here, meaning column in the original non-tensor form matrix). This reduces the algorithmic complexity from $O(\alpha \prod_{c=1}^{C} \Lambda_c^2)$ to $O\left(\alpha\left(\prod_{c=1}^{C} \Lambda_c\right)\left(\sum_{c=1}^{C} \Lambda_c\right)\right)$. We note that while the extraction of the Edman degradation tensor appears to have little direct effect on the algorithmic complexity reduction, which is because it has a sparsity effect on the original transition tensor, properly handling Edman degradation is critical to this decomposition. We feel this is the easiest way to do this while also factoring the fluorophore loss effects into separate tensors.

Everything all together

Handling peptide detachment is simpler. We modify $\boldsymbol{\mathcal{T}}$ to be:

$$\boldsymbol{\mathcal{T}}_{\boldsymbol{vjui}} = \begin{cases} (1-d)e\beta(\boldsymbol{i},\boldsymbol{j},p), & \text{if } \boldsymbol{j} \leq \boldsymbol{i} \text{ and } v = u \\ (1-d)(1-e)\beta(\boldsymbol{i},\boldsymbol{j}), & \text{if } \boldsymbol{j} \leq \boldsymbol{i} \text{ and } v = u+1 \text{ and } \bar{c}_u = 0 \\ (1-d)(1-e)\left(\left(1-\frac{i_{\bar{c}_u}}{\lambda_u}\right)\beta(\boldsymbol{i},\boldsymbol{j}) + \left(\frac{i_{\bar{c}_u}}{\lambda_u}\right)\bar{\beta}(\boldsymbol{i},\boldsymbol{j},u)\right), & \text{if } \boldsymbol{j} \leq \boldsymbol{i} \text{ and } v = u+1 \text{ and } \bar{c}_u > 0 \\ d, & \text{if } j_c = 0 \forall c \text{ and } v = \alpha \\ 0, & \text{otherwise} \end{cases} \tag{24}$$

This creates a new "empty" state which can always be transitioned to with probability d of detachment. The probability of avoiding this state is $(1-d)$. The functions β and $\bar{\beta}$ are the same as before in (19) and (20). The matrix vector multiplication step of the HMM forward algorithm has not changed from (17). We can then construct a new tensor $\boldsymbol{\mathcal{D}}$ for peptide detachment which satisfies:

$$\boldsymbol{\mathcal{D}}_{\boldsymbol{whvk}} = \begin{cases} 1-d, & \text{if } \boldsymbol{h} = \boldsymbol{k} \text{ and } w = v \leq \alpha \\ d & \text{if } h_c = 0 \forall c \text{ and } w = \alpha + 1 \end{cases} \quad (25)$$

Then we find that:

$$\boldsymbol{\mathcal{T}}_{\boldsymbol{wlui}} = \boldsymbol{\mathcal{D}}_{\boldsymbol{wlvk}} \boldsymbol{\mathcal{E}}_{\boldsymbol{vkuj}} \boldsymbol{\mathcal{B}}^{(1)}_{j_1 i_1} \boldsymbol{\mathcal{B}}^{(2)}_{j_2 i_2} \dots \boldsymbol{\mathcal{B}}^{(C)}_{j_C i_C} \quad (26)$$

Substituting into (17) with another multi-index $\boldsymbol{m} = m_1 m_2 \dots m_C$ provides:

$$\boldsymbol{f}^{(t+1)}_{\boldsymbol{wm}} = \boldsymbol{\mathcal{O}}^{(t+1)}_{\boldsymbol{ml}} \boldsymbol{\mathcal{D}}_{\boldsymbol{wlvk}} \boldsymbol{\mathcal{E}}_{\boldsymbol{vkuj}} \boldsymbol{\mathcal{B}}^{(1)}_{j_1 i_1} \boldsymbol{\mathcal{B}}^{(2)}_{j_2 i_2} \dots \boldsymbol{\mathcal{B}}^{(C)}_{j_C i_C} \boldsymbol{f}^{(t)}_{\boldsymbol{ui}} \quad (27)$$

$\boldsymbol{\mathcal{D}}$ is clearly highly sparse, with two entries in each column of the original matrix in non-tensor form. Thus, $\boldsymbol{\mathcal{D}}$ has no impact on the algorithmic complexity of this operation. Although $\boldsymbol{\mathcal{D}}$ and $\boldsymbol{\mathcal{E}}$ could be combined to achieve this same algorithmic improvement, we found that this separation made our model easier to reason about and work with.

Transition matrix factoring conclusions

One of the benefits of this approach to algorithmic complexity reduction is that this factorization provides no loss to the theoretical accuracy of the forward algorithm. No theoretical approximations were necessary, aside from the unavoidable differences in floating-point round-off errors. This allows for highly accurate results with much more efficient runtime characteristics than a naïve implementation.
