# Supplementary material for: Amino acid sequence assignment from single molecule peptide sequencing data using a two-stage classifier
Source: PLoS Comput Biol. 2023 May 30;19(5):e1011157. doi: 10.1371/journal.pcbi.1011157 (PMC10256185; doi:10.1371/journal.pcbi.1011157)
Supplement: S3 Appendix — (PDF) [file pcbi.1011157.s004.pdf]

HMM pruning

Because the emission matrix is diagonal, it is equivalent to the diagonal part of its Singular Value Decomposition (SVD), but with a reordering of its indices. This makes sparsification of this matrix equivalent to the Eckart-Young-Mirsky theorem; we can keep the largest r values for some chosen value of r, and replace the rest of the matrix entries with zeros, having the minimum possible impact on the spectral and Frobenius norms for the chosen value of r.

Furthermore, we can propagate this sparsification to the transition matrix. Consider the forward algorithm, with $\boldsymbol{T}$ representing the transition matrix, and $\boldsymbol{O}^{(t)}$ representing the diagonal emission matrix for time t. Then if $\boldsymbol{f}^{(t)}$ represents the vector of intermediate probabilities at time t, we have:

$$\boldsymbol{f}^{(t+1)} = \boldsymbol{O}^{(t+1)}\boldsymbol{T}\boldsymbol{f}^{(t)} \tag{28}$$

Now we sparsify each $\boldsymbol{O}^{(t)}$ as discussed above, to get a series of $\widehat{\boldsymbol{O}}^{(t)}$. This gives:

$$\boldsymbol{f}^{(t+1)} = \widehat{\boldsymbol{O}}^{(t+1)}\boldsymbol{T}\boldsymbol{f}^{(t)} \tag{29}$$

Note that we have many copies of $\boldsymbol{T}$, which are equal. For our next improvements we need these to be different for each timestep, so we can rewrite (29) with $\boldsymbol{T}^{(t)}$ for each timestep t, giving

$$\boldsymbol{f}^{(t+1)} = \widehat{\boldsymbol{O}}^{(t+1)}\boldsymbol{T}^{(t)}\boldsymbol{f}^{(t)} \tag{30}$$

Here the values of many rows and columns of each $\boldsymbol{T}^{(t)}$ have been made unnecessary by the sparsification of its neighboring $\widehat{\boldsymbol{O}}^{(t+1)}$ and $\widehat{\boldsymbol{O}}^{(t)}$, as any vector product with $\widehat{\boldsymbol{O}}^{(t)}$ will necessarily have zeros except for the r entries retained, such that we need only keep the corresponding r columns of $\boldsymbol{T}^{(t)}$. Similarly, any entry in the vector product with $\boldsymbol{T}^{(t)}$ which is not multiplied by one of the r entries retained in $\widehat{\boldsymbol{O}}^{(t+1)}$ is multiplied by zeros, and is thus unnecessary, so we need only keep the corresponding r rows of $\boldsymbol{T}^{(t)}$. Calling these approximations $\widehat{\boldsymbol{T}}^{(t)}$, we get

$$\boldsymbol{f}^{(t+1)} = \widehat{\boldsymbol{O}}^{(t+1)}\widehat{\boldsymbol{T}}^{(t)}\boldsymbol{f}^{(t)} \tag{31}$$

This allows significant sparsity to be used (Fig 5). Previously this formula would have been $O(\alpha^2 T \prod_{c=1}^{C} \Lambda_c^2)$ to compute repeatedly across all timesteps, while this reduces the algorithmic complexity to $O((r^2 + \alpha(\prod_{c=1}^{C} \Lambda_c) \log r)T)$. Here $\alpha(\prod_{c=1}^{C} \Lambda_c) \log r$ represents the algorithmic cost of determining the r largest elements on the diagonal of the emission matrix using a priority queue. This improvement is beyond what is possible in a more traditional usage of sparse matrix multiplication. For sparse matrix multiplication, we would need to first multiply $\widehat{\boldsymbol{T}}^{(t)}$ by $\widehat{\boldsymbol{O}}^{(t)}$ or multiply $\widehat{\boldsymbol{O}}^{(t+1)}$ by $\widehat{\boldsymbol{T}}^{(t)}$. This will only permit you to sparsify your operations on the rows or the columns of $\boldsymbol{T}$ but not both, giving a complexity of $O(r\alpha T \prod_{c=1}^{C} \Lambda_c)$ (here sorting considerations are dominated by the rest of the formula and can be omitted). While this is better than not using this inherent sparsity at all, preprocessing the transition matrix in consideration of the emission matrices on either side gives better results in algorithmic complexity.

In practice, we use a more complicated pruning scheme, as detailed in S4 Appendix.
