# Supplementary material for: Amino acid sequence assignment from single molecule peptide sequencing data using a two-stage classifier
Source: PLoS Comput Biol. 2023 May 30;19(5):e1011157. doi: 10.1371/journal.pcbi.1011157 (PMC10256185; doi:10.1371/journal.pcbi.1011157)
Supplement: S4 Appendix — (PDF) [file pcbi.1011157.s005.pdf]

Combining transition matrix factoring with HMM pruning

By making r suitably small, HMM pruning can exhibit better algorithmic complexity than if we factor the transition matrix. However, we believe it is much better to combine these algorithmic enhancements (Fig 6). To do this, we need to switch into tensor notation, replacing our matrices and vectors with the tensor equivalents we constructed previously. This yields:

$$\boldsymbol{f}_{\boldsymbol{vk}}^{(t+1)} = \hat{\boldsymbol{\mathcal{O}}}_{\boldsymbol{kj}}^{(t+1)} \hat{\boldsymbol{\mathcal{T}}}_{\boldsymbol{vjui}}^{(t)} \boldsymbol{f}_{\boldsymbol{ui}}^{(t)} \tag{32}$$

We also want to use the factorization from (26), using timestamp specific sub-tensors of each of the factored pieces. The factorization of (26) becomes:

$$\hat{\boldsymbol{\mathcal{T}}}_{\boldsymbol{wlui}}^{(t)} = \hat{\boldsymbol{\mathcal{D}}}_{\boldsymbol{wlvk}}^{(t)} \hat{\boldsymbol{\mathcal{E}}}_{\boldsymbol{vkuj}}^{(t)} \hat{\boldsymbol{\mathcal{B}}}_{j_1 i_1}^{(t,1)} \hat{\boldsymbol{\mathcal{B}}}_{j_2 i_2}^{(t,2)} \dots \hat{\boldsymbol{\mathcal{B}}}_{j_C i_C}^{(t,C)} \tag{33}$$

Substituting into (32) gives:

$$\boldsymbol{f}_{\boldsymbol{wm}}^{(t+1)} = \hat{\boldsymbol{\mathcal{O}}}_{\boldsymbol{ml}}^{(t+1)} \hat{\boldsymbol{\mathcal{D}}}_{\boldsymbol{wlvk}}^{(t)} \hat{\boldsymbol{\mathcal{E}}}_{\boldsymbol{vkuj}}^{(t)} \hat{\boldsymbol{\mathcal{B}}}_{j_1 i_1}^{(t,1)} \hat{\boldsymbol{\mathcal{B}}}_{j_2 i_2}^{(t,2)} \dots \hat{\boldsymbol{\mathcal{B}}}_{j_C i_C}^{(t,C)} \boldsymbol{f}_{\boldsymbol{ui}}^{(t)} \tag{34}$$

Suppose we were to use standard sparse tensor multiplication techniques and carry this operation out from right to left. Each tensor $\hat{\boldsymbol{\mathcal{B}}}_{j_c i_c}^{(t,c)}$ can introduce any entry of input (index i_c) into as many as Λ_c indices of output (index j_c). The resulting computational complexity of the forward algorithm, even with the given sparsity, is then $O\left(r\alpha T \prod_{c=1}^{C} \Lambda_c\right)$.

If we preprocess the computation, pruning each operation now from both directions, the algorithmic complexity does not improve the way it does in the matrix case, although likely this would behave faster in practice. The problem is that the pruning operation itself needs to determine which rows to propagate forwards, which requires accessing every non-zero entry reachable in the forward direction. Many of these values are later pruned in the backwards direction, so the computation itself has much better sparsity, but the time to prune then dominates the algorithmic complexity result.

To improve this further, we add structure to the pruning of $\widehat{\boldsymbol{\mathcal{O}}}^{(t)}$. Instead of keeping the r largest values in $\widehat{\boldsymbol{\mathcal{O}}}^{(t)}$, we prune each index of $\widehat{\boldsymbol{\mathcal{O}}}^{(t)}$ independently. For additional convenience, we limit each index to a contiguous range of values. Then we let each index for any fluorophore color c have r_c values and allow $\bar{r}$ values to index the number of amino acids. These simplifications may cause the pruning to be non-optimal, but we accept this trade-off.

We can then prune our tensors using their known structures (for example, the tensors $\widehat{\boldsymbol{\mathcal{B}}}^{(t,c)}$ correspond to an upper triangular matrix). This time when we propagate the pruning results in both directions, the time required is only $O(C^2)$ (the number of minimum and maximum indices to be propagated through each tensor scales with C, as does the number of tensors to be pruned). For the runtime of the tensor operations, consider each tensor individually. $\widehat{\boldsymbol{\mathcal{D}}}^{(t)}$ and $\widehat{\boldsymbol{\mathcal{E}}}^{(t)}$ are both highly sparse, so they contribute a constant modification to the number of rows or columns when propagating in either direction. $\widehat{\boldsymbol{\mathcal{D}}}^{(t)}$ requires special handling. We track the detached state separately from the ordinary range, to avoid unnecessarily including a large range of states which don't need to be.

Then each $\widehat{\boldsymbol{\mathcal{B}}}^{(t,c)}$ operates on an independent index, and therefore can be considered on its own. This tensor after pruning will have dimensions that are $O(r_c^2)$, and should have a constant effect on the number of elements input vs output. Therefore, each of these tensors will require an algorithmic complexity of $O\left(r_c \bar{r} \prod_{\tilde{c}=1}^{C} r_{\tilde{c}}\right)$. Bringing this all together we get an algorithmic complexity of $O\left(C^2 + \bar{r}\left(\sum_{c=1}^{C} r_c\right)\left(\prod_{c=1}^{C} r_c\right)\right)$ for processing one timestep. The full forward algorithm then has a complexity of $O\left(T\left(C^2 + \bar{r}\left(\sum_{c=1}^{C} r_c\right)\left(\prod_{c=1}^{C} r_c\right)\right)\right)$.

One remaining clarification is the manner of choosing r_c and $\bar{r}$. In fact, these values should not be kept constant; let us refer to the values for time t as $r_c^{(t)}$ and $\bar{r}^{(t)}$. $\bar{r}^{(0)} = 1$ and $\bar{r}^{(t+1)} = \bar{r}^{(t)} + 1$, due to the possibility of amino acid removal. These on average are proportional to α. To get $r_c^{(t)}$, we keep all index

values where a fluorophore count of that value has the observed fluorescence intensity for color c at time t within a specified confidence interval – perhaps within 3σ of the mean, where σ is the standard deviation of the distribution. These will necessarily be contiguous. The number of indices kept is then $r_c^{(t)}$.

The standard deviation of a normal distribution scales with the square root of the intensity, and the number of possible index values is limited by the total possible number of fluorophores of color c. It follows that any removal of index values proportional to the standard deviation will satisfy $r_c^{(t)} < \gamma\sqrt{\Lambda_c}$ for some constant γ dependent on the cutoff. Then the algorithmic complexity is given by

$$O\left(T\left(C^2 + \alpha\left(\sum_{c=1}^{C}\sqrt{\Lambda_c}\right)\left(\prod_{c=1}^{C}\sqrt{\Lambda_c}\right)\right)\right).$$

We chose a specific pruning cut-off by sweeping this parameter and balancing the experimental runtime effects and the precision-recall curves which result from simulated data.
