# Supplementary figures and images for: Amino acid sequence assignment from single molecule peptide sequencing data using a two-stage classifier
Source: PLoS Comput Biol. 2023 May 30;19(5):e1011157. doi: 10.1371/journal.pcbi.1011157 (PMC10256185; doi:10.1371/journal.pcbi.1011157)

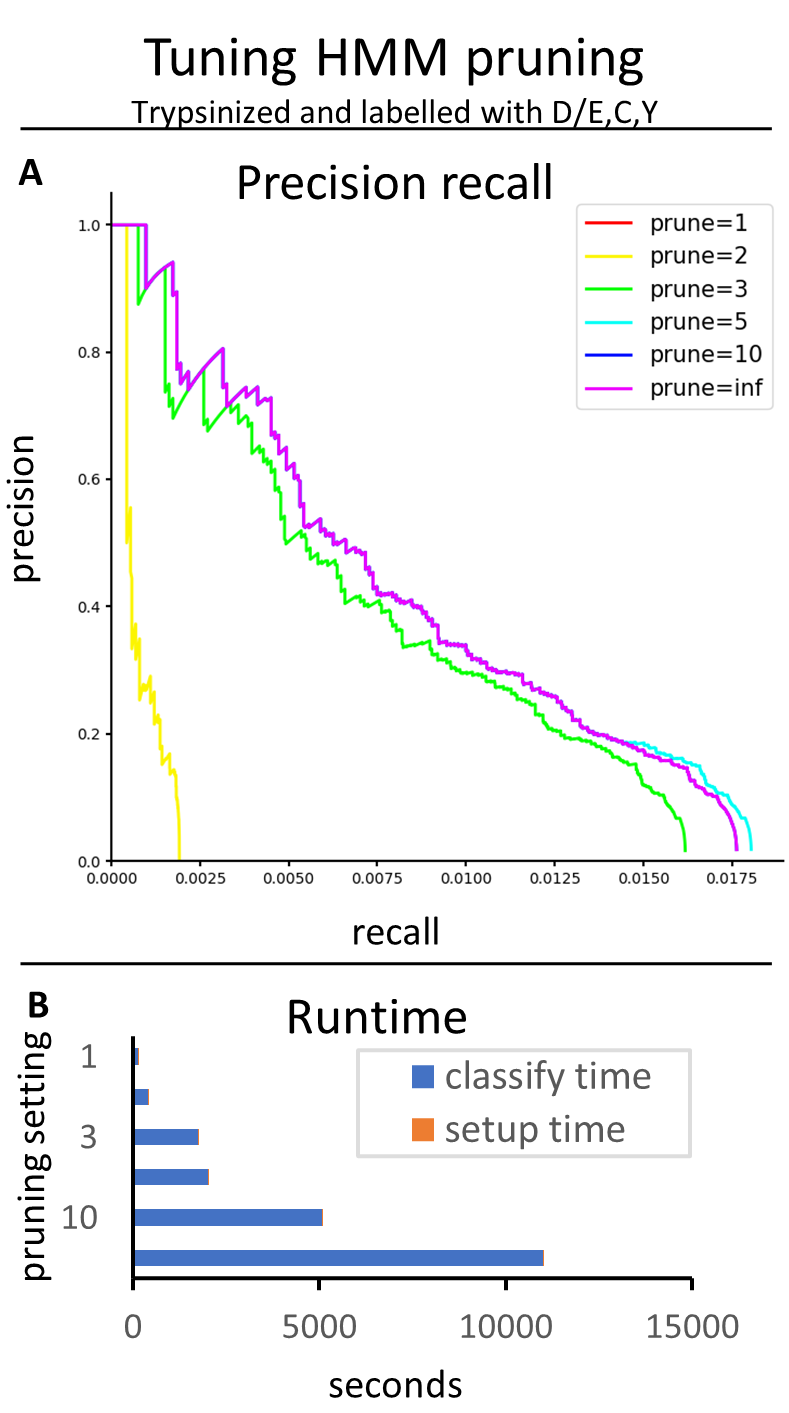

Supplement: S1 Fig — Setting this parameter to 5 (i. e., 5 σ) appears to provide the best trade-off. (A) Precision/recall curves. We note that “prune = 5” and “prune = 10” are hidden under the “prune = inf” curve. The “prune = 1” curve contains no true positives. (B) Runtimes. (TIF) [file pcbi.1011157.s006.tif]

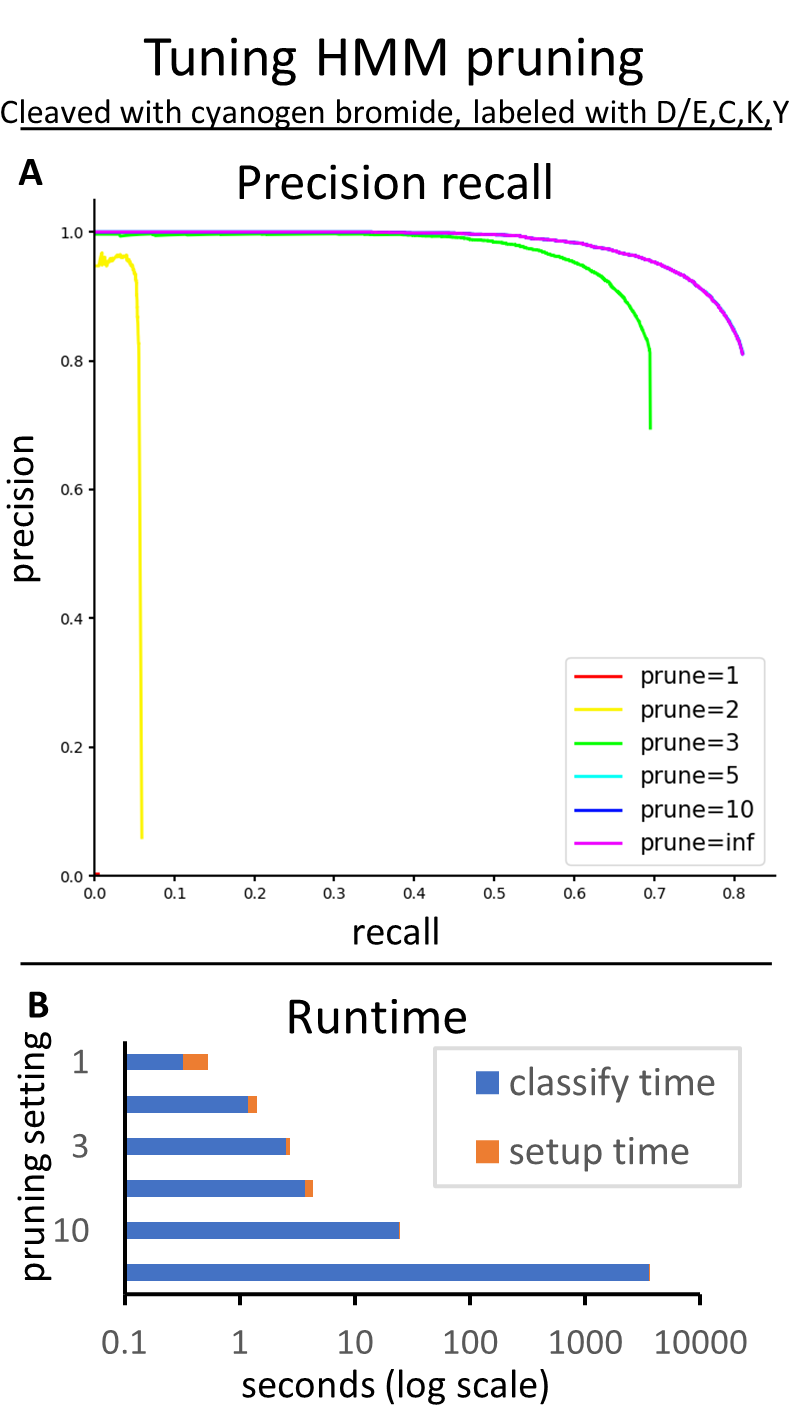

Supplement: S2 Fig — Here we show a more extraordinary case than S1 Fig. (A) Precision/recall curves. We note that “prune = 5” and “prune = 10” are hidden under the “prune = inf” curve. The “prune = 1” curve contains no true positives. (B) Runtimes. (TIF) [file pcbi.1011157.s007.tif]

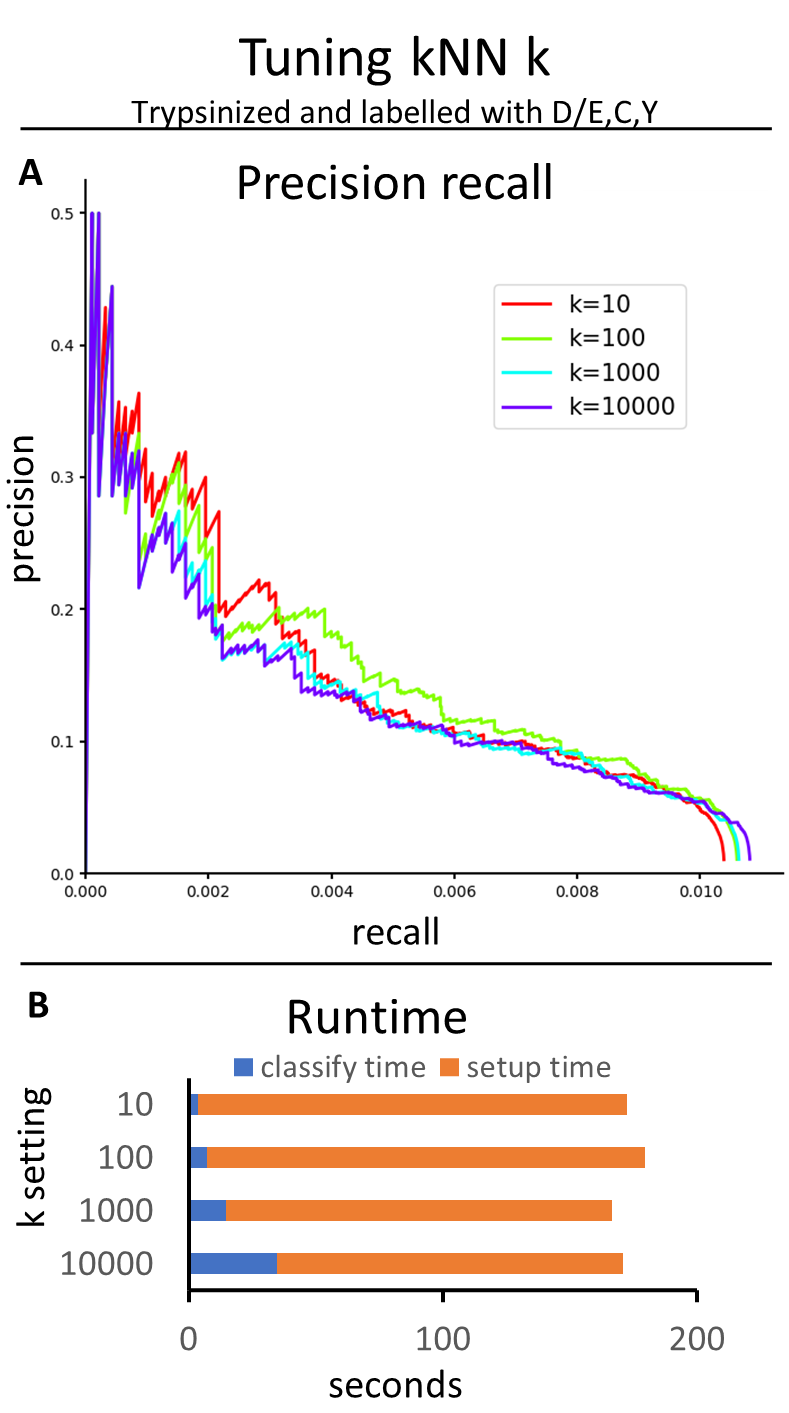

Supplement: S3 Fig — Setting k to 10 seems to provide the best trade-off (A) Precision/recall curves. (B) Runtimes. (TIF) [file pcbi.1011157.s008.tif]

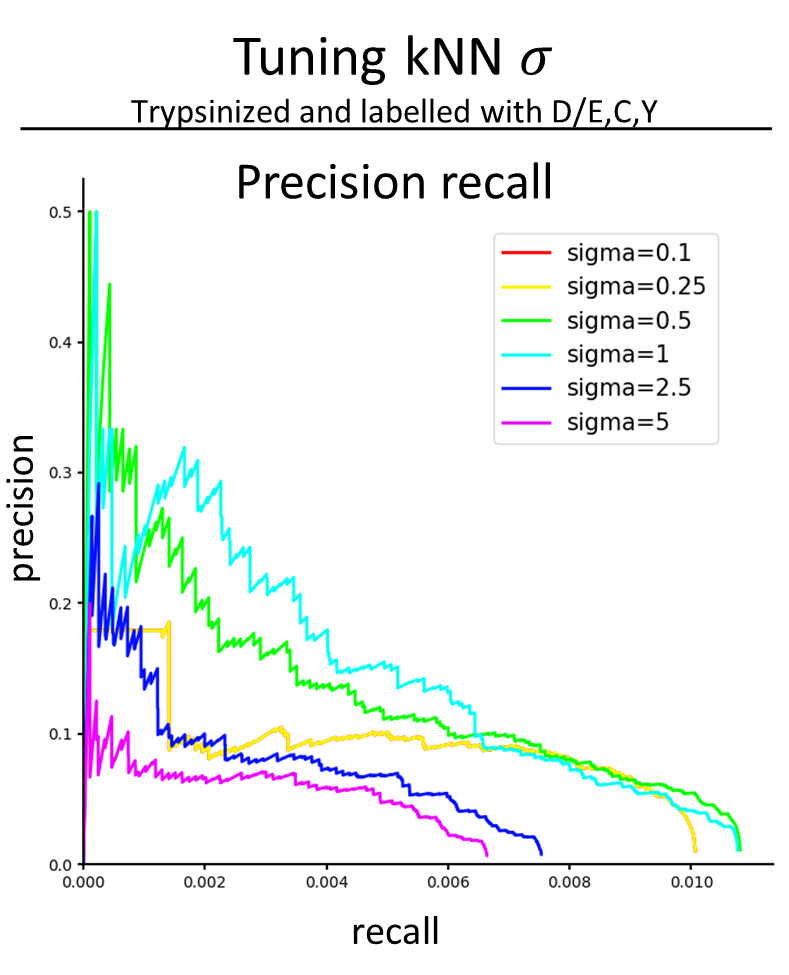

Supplement: S4 Fig — Setting σkNN to 0.5 seems to provide the best trade-off. All settings showed a classify time of about 35 seconds, and a setup time of about 140 seconds. (TIF) [file pcbi.1011157.s009.tif]

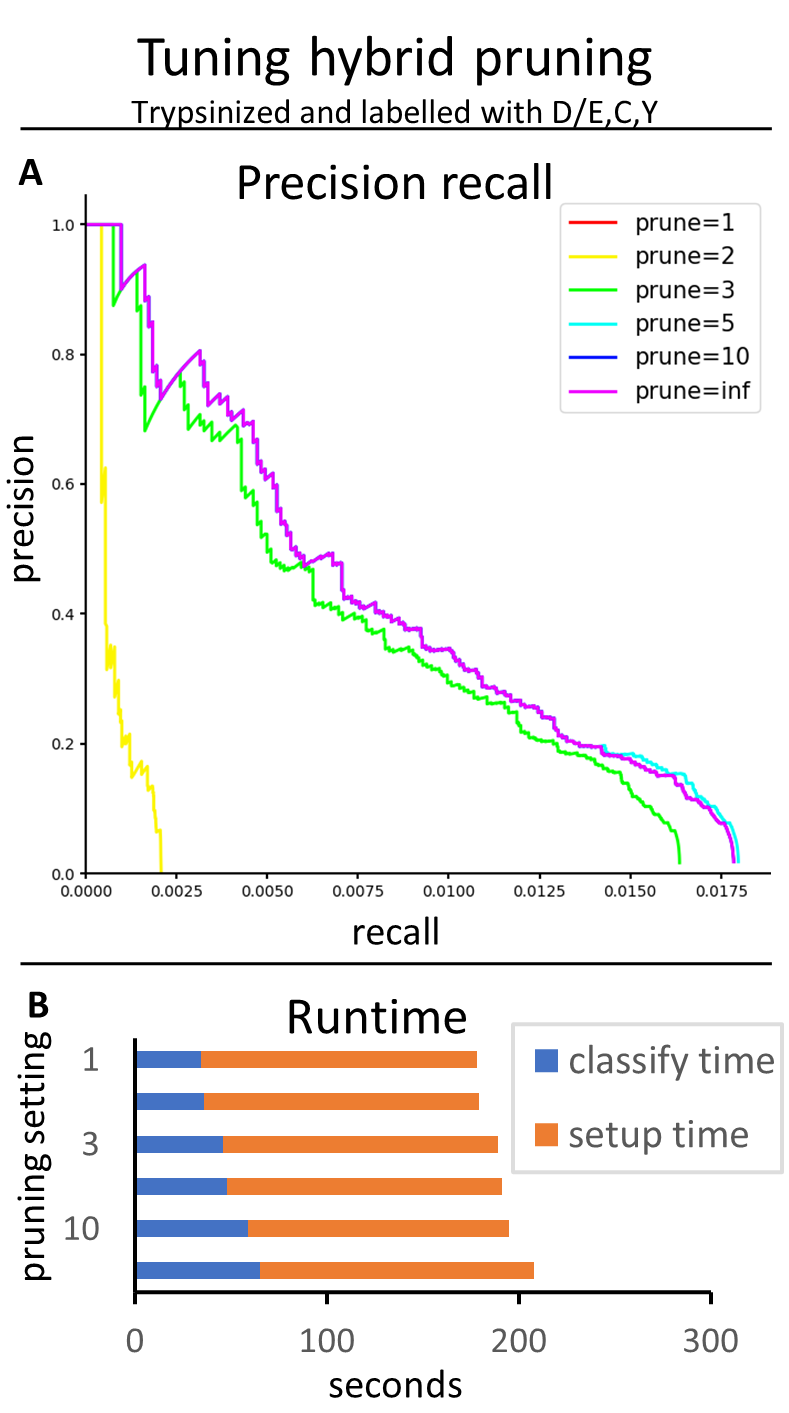

Supplement: S5 Fig — A value of 5 appeared to provide the best trade-off. (A) Precision/recall curves. We note that “prune = 5” and “prune = 10” are hidden under the “prune = inf” curve. The “prune = 1” curve contains no true positives. (B) Runtimes. (TIF) [file pcbi.1011157.s010.tif]

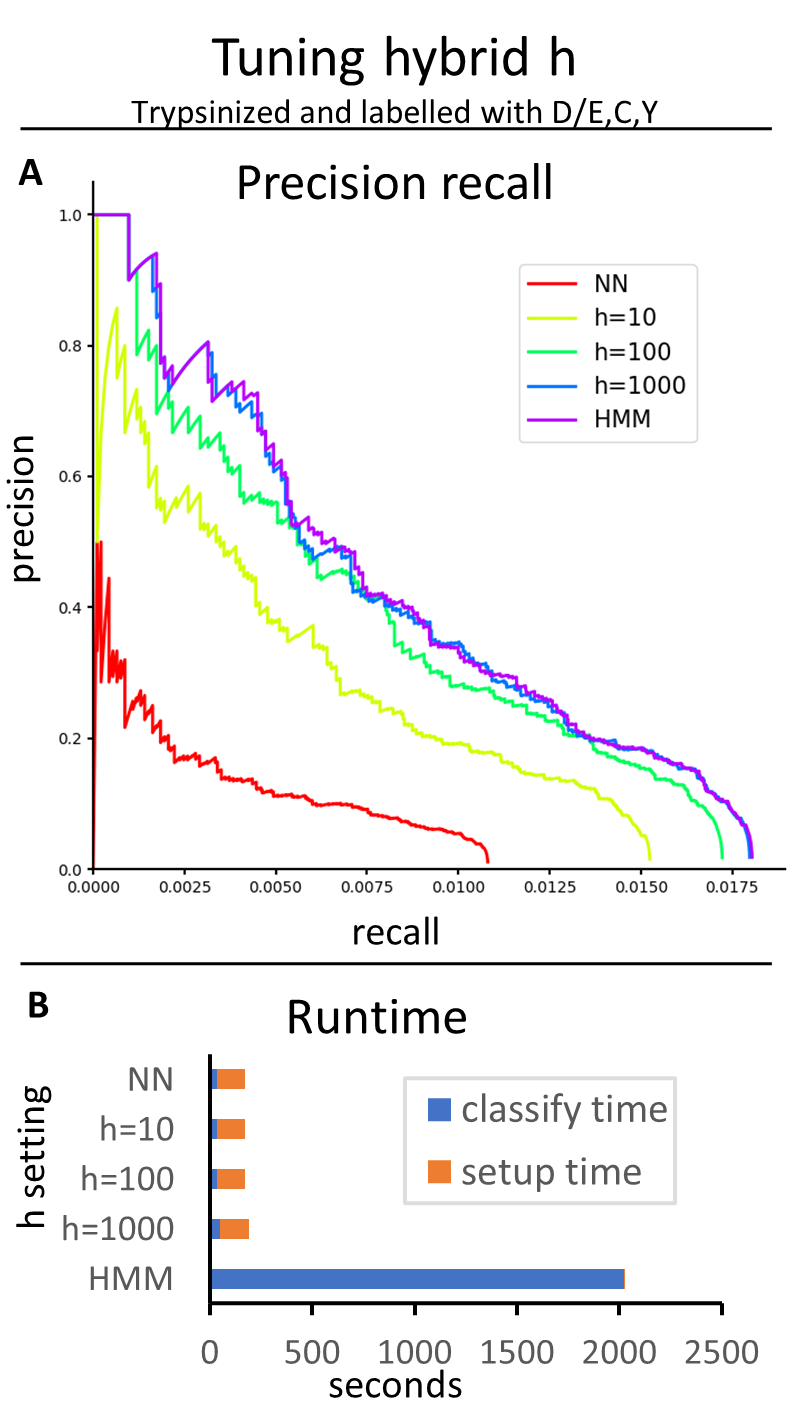

Supplement: S6 Fig — An h of 1000 provided the best trade-off. (A) Precision/recall curves. (B) Runtimes. (TIF) [file pcbi.1011157.s011.tif]

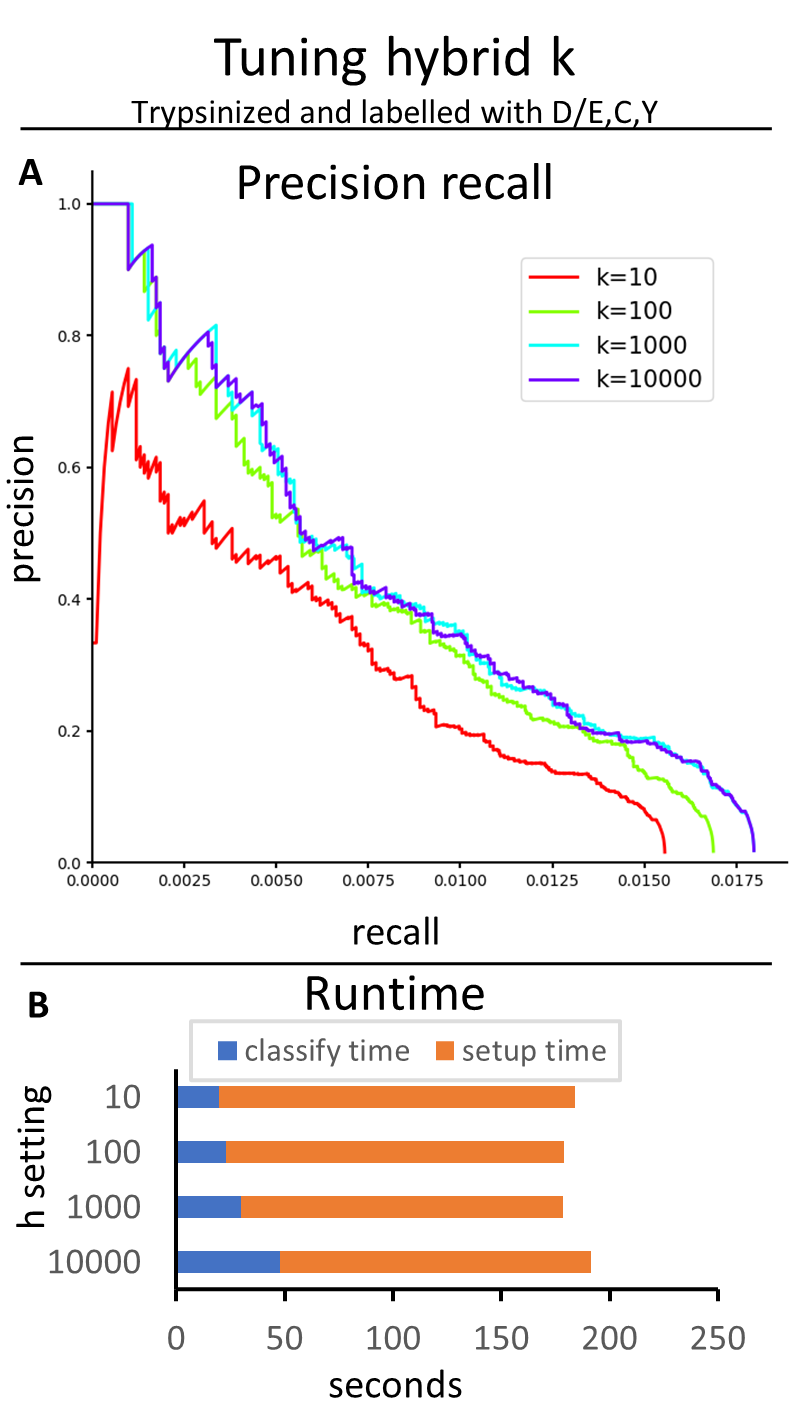

Supplement: S7 Fig — A k of 1000 or 10000 provided the best results. (A) Precision/recall curves. (B) Runtimes. (TIF) [file pcbi.1011157.s012.tif]

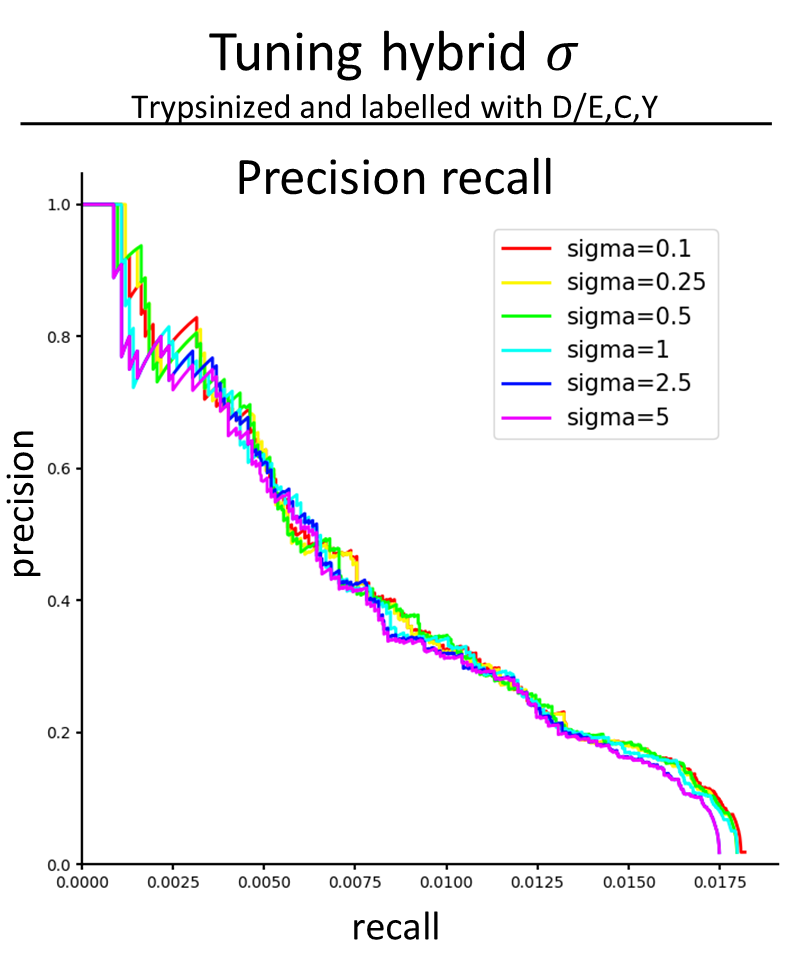

Supplement: S8 Fig — Setting σ to 0.5 seemed to provide the best trade-off. All settings had a classify time of about 50 seconds, and a setup time of about 140 seconds. (TIF) [file pcbi.1011157.s013.tif]

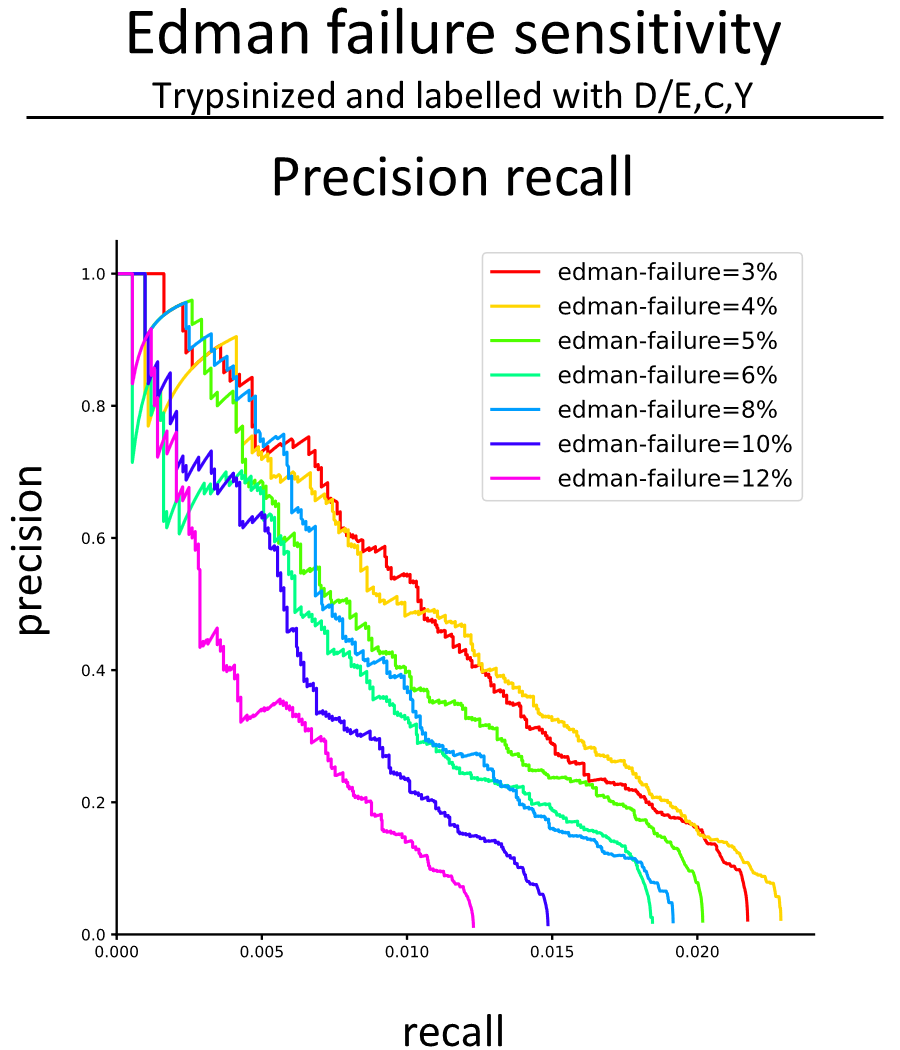

Supplement: S9 Fig — The model expects an Edman failure rate of 6%, the rate for the test data varies as in the legend. Run on a full human proteome dataset. (TIF) [file pcbi.1011157.s014.tif]

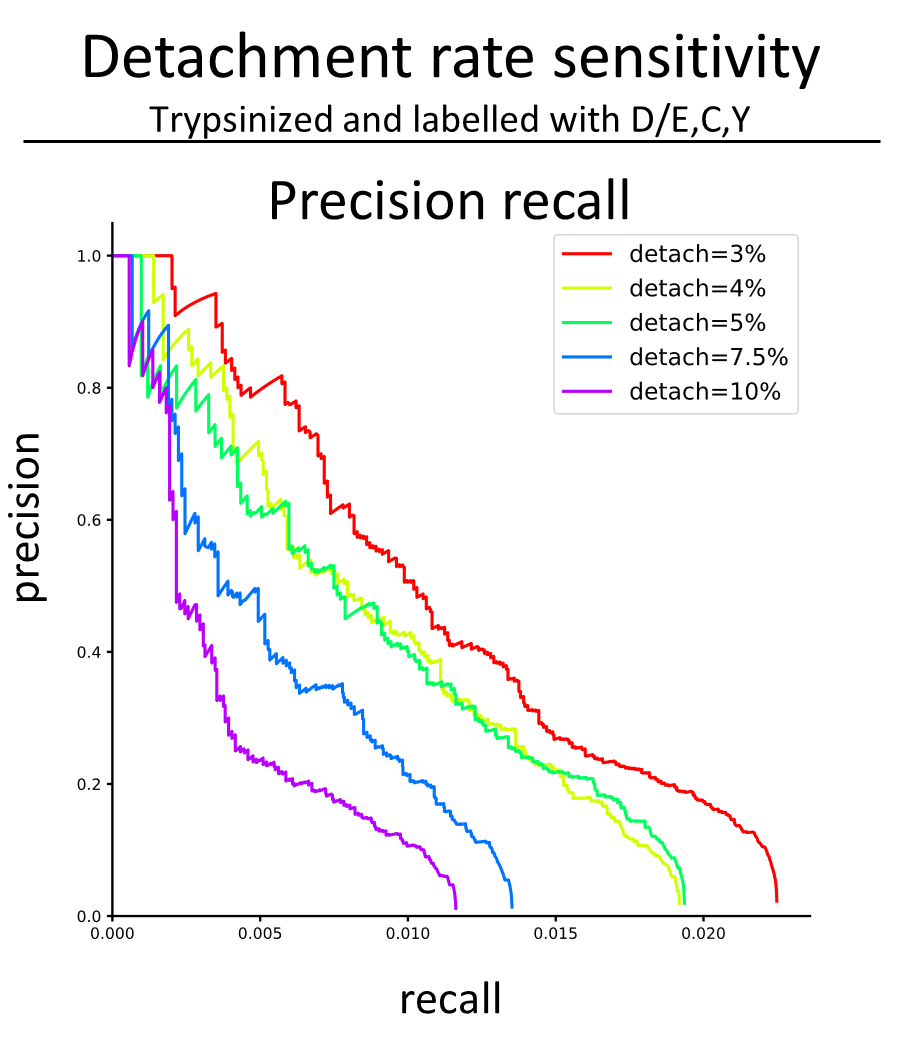

Supplement: S10 Fig — The model expects a detachment rate of 5%, the rate for the test data varies as in the legend. Run on a full human proteome dataset. (TIF) [file pcbi.1011157.s015.tif]

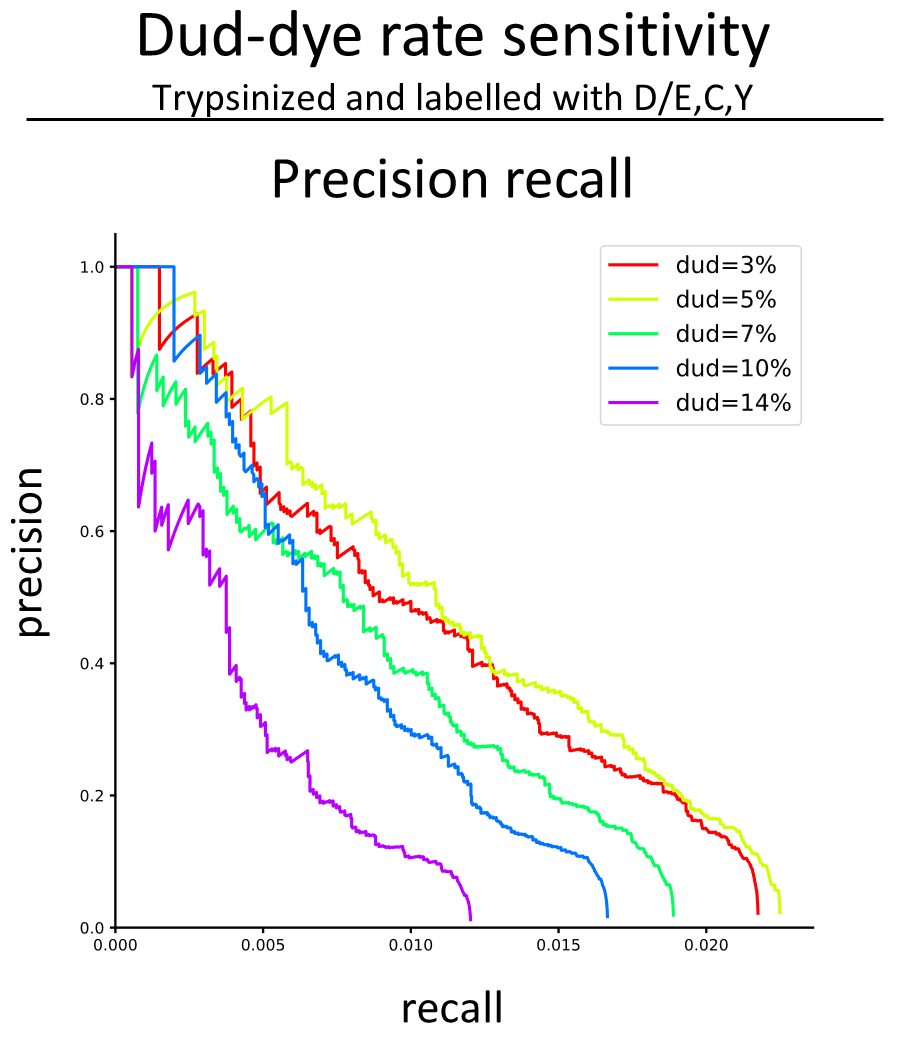

Supplement: S11 Fig — The model expects a dud-dye rate of 7%, the rate for the test data varies as in the legend. Run on a full human proteome dataset. (TIF) [file pcbi.1011157.s016.tif]

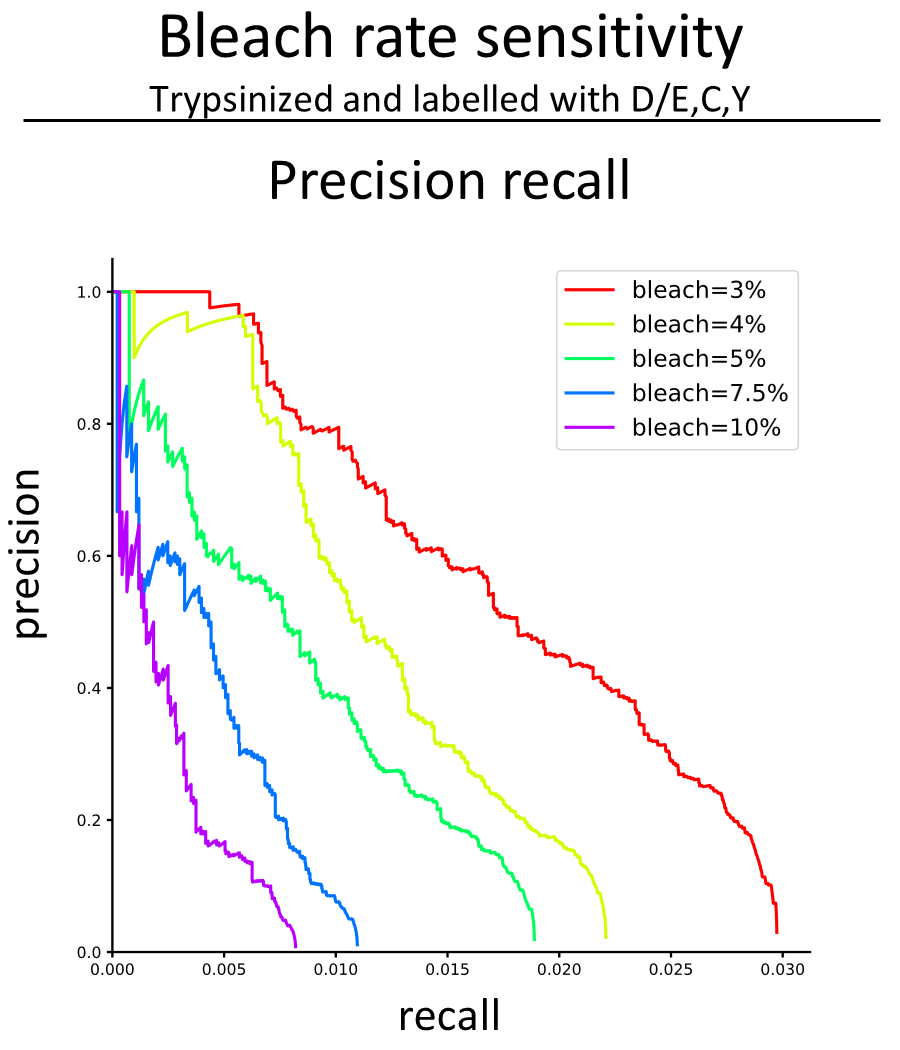

Supplement: S12 Fig — The model expects a bleach rate of 5%, the rate for the test data varies as in the legend. Run on a full human proteome dataset. (TIF) [file pcbi.1011157.s017.tif]

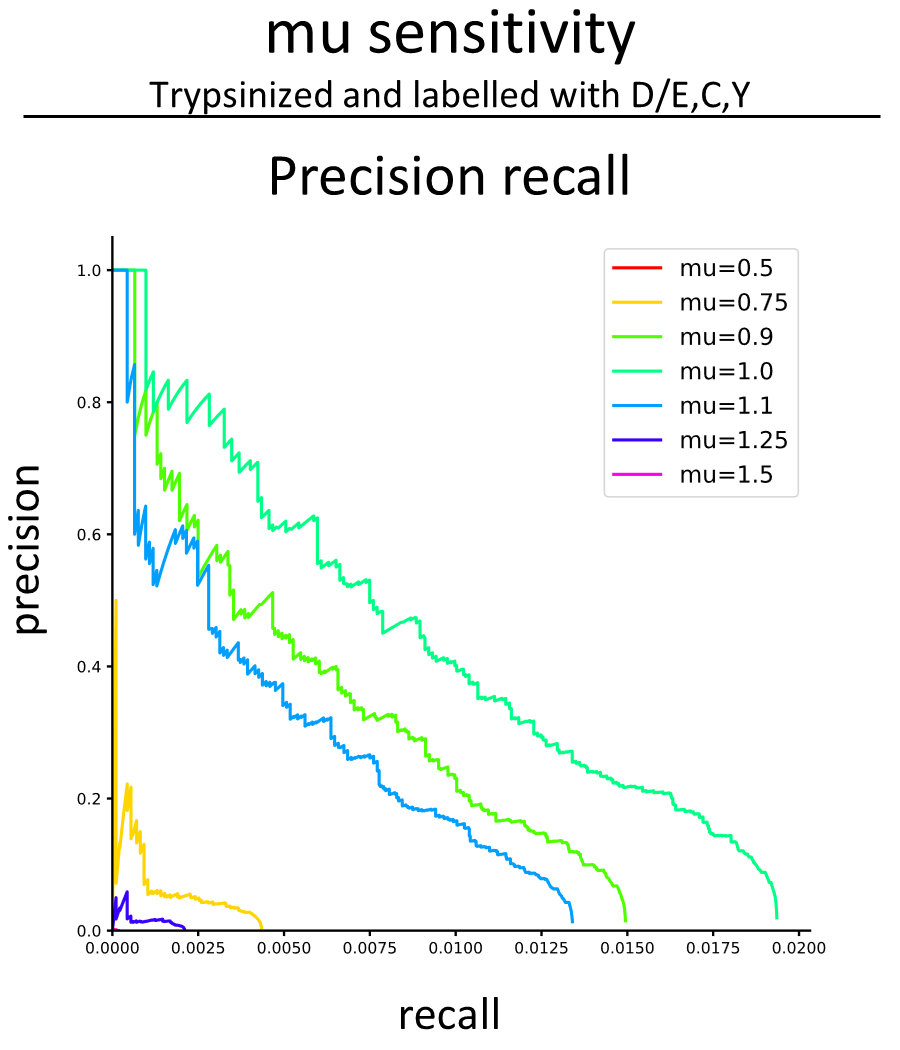

Supplement: S13 Fig — The model expects a mu value of 1.0, the rate for the test data varies as in the legend. Run on a full human proteome dataset. (TIF) [file pcbi.1011157.s018.tif]

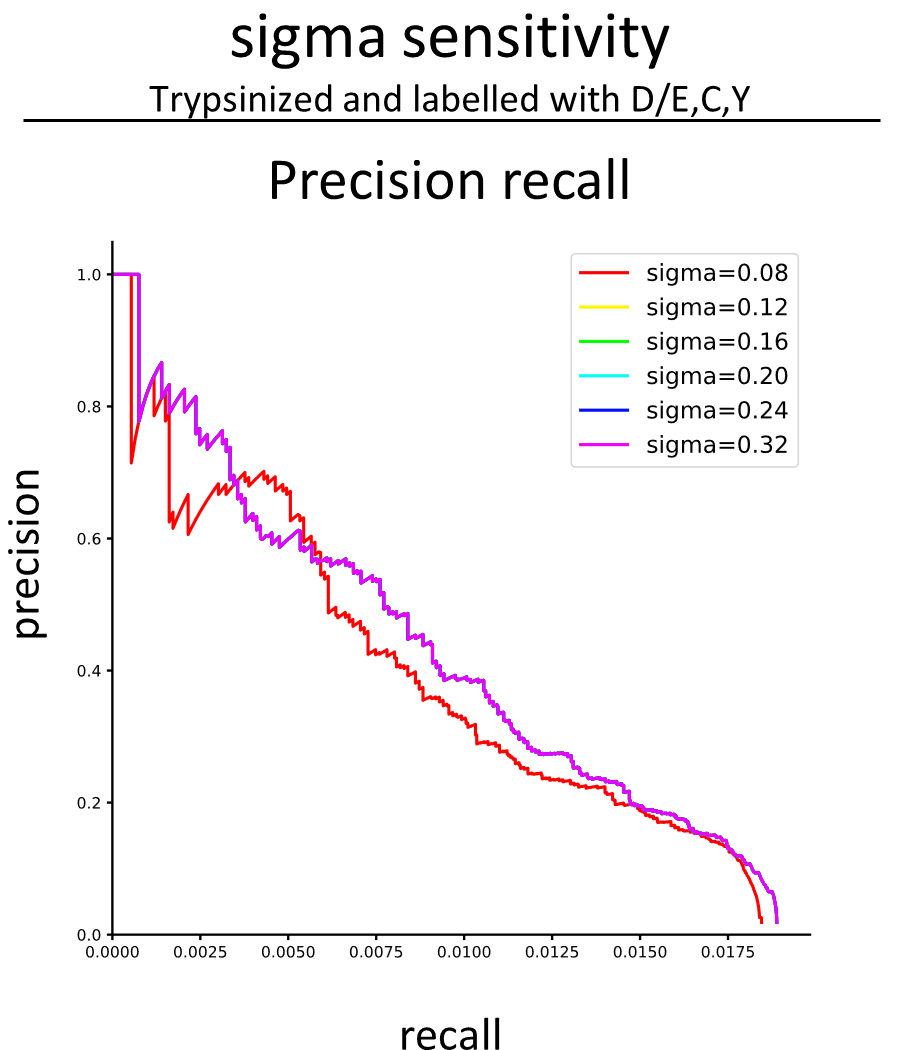

Supplement: S14 Fig — The model expects a sigma value of .16, the rate for the test data varies as in the legend. Run on a full human proteome dataset. (TIF) [file pcbi.1011157.s019.tif]

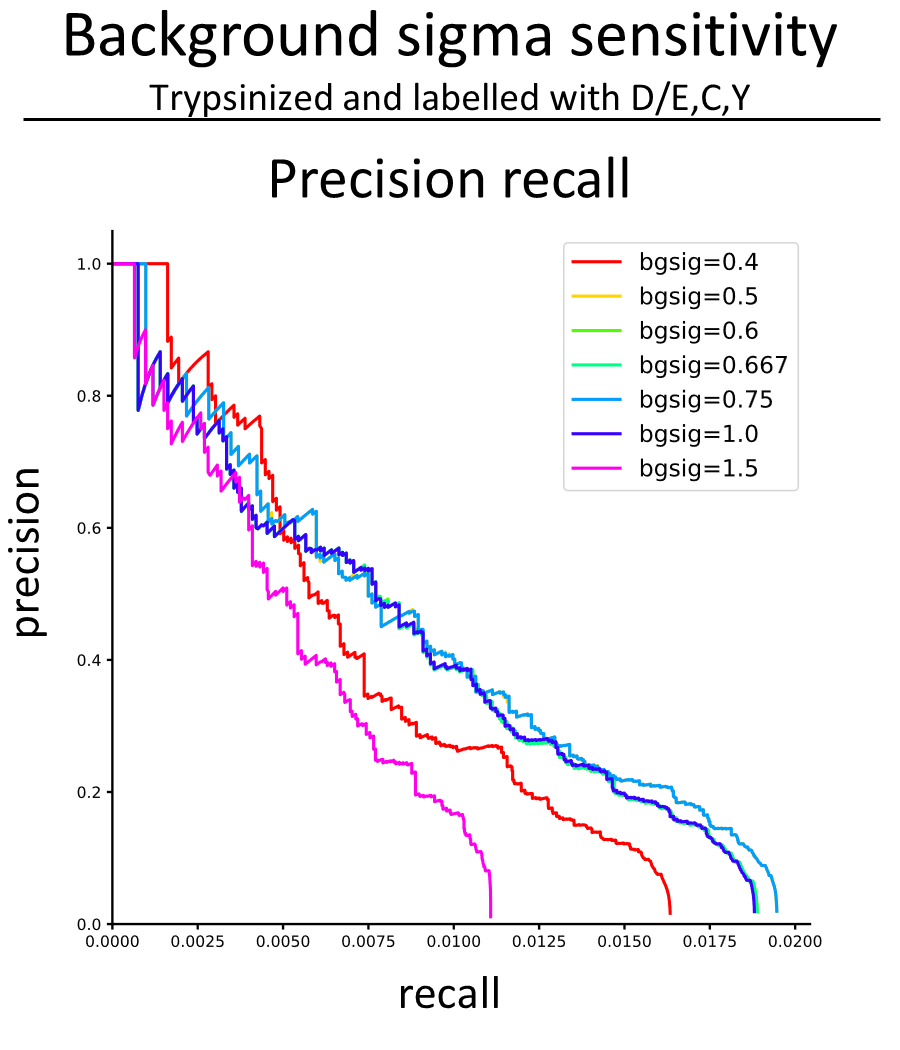

Supplement: S15 Fig — The model expects a background sigma value of .00667, the rate for the test data varies as in the legend. Run on a full human proteome dataset. (TIF) [file pcbi.1011157.s020.tif]
