# Supplementary material for: Amino acid sequence assignment from single molecule peptide sequencing data using a two-stage classifier
Source: PLoS Comput Biol. 2023 May 30;19(5):e1011157. doi: 10.1371/journal.pcbi.1011157 (PMC10256185; doi:10.1371/journal.pcbi.1011157)
Supplement: Attachment — Submitted filename: whatprot paper - revisions_responses-1.pdf [file pcbi.1011157.s021.pdf]

Responses to referees for PCOMPBIOL-D-23-00097
Smith *et al.*, Amino acid sequence assignment from single molecule peptide sequencing data using a two-stage classifier

Our responses follow in-line in blue text.

Reviewer #1: The paper proposes a two-stage peptide classification algorithm for data from florosequencing. The two stages consist of a k-nearest neighbor (kNN) classifier that can select a moderate number of candidate sequences whose data likelihood can be (approximately) computed by the second step using a hidden Markov Model for the data and turned into approximate posterior sequence probabilities by Bayes rule and summing of the kNN candidate sequences.

The paper is excellent. The idea is good and well-explained, the results are impressive, and the writing is impeccable. The paper can be accepted in its current form, and I have embarrassingly little feedback to give. A few short notes are provided below in the order in which the points appear in the paper, but I would like to leave it up to the authors' discretion what (if any) changes to make due to these, as most of them are mainly stylistic.

We thank the referee for their extremely positive assessment of our paper. We have addressed each of the specific comments below.

Line 104: "0.16" -> ".16" to match the prior style?

Done.

Line 120: "Each fluorophore count is stored as a two-byte numeric value." I found this note overly specific and think that it can be removed. It is an implementation detail in a section that is about concepts. The same comment applies to line 124 about double-precision floating-point values.

We removed these sentences.

Line 138: Why you use the word "approximated" here is unclear. I understand the approximations introduced later, but here you are describing the exact transition probabilities the model gives. Do you mean that the model itself is an approximation? Consider reformulating this statement.

We change “approximated” to “calculated” here since the probabilities are simply calculated directly from the experimentally derived error rates.

Line 150: To be a bit pedantic, (2) should not be a *probability* given that Y is a continuous

random variable. Maybe this can be dealt with by simply adding a footnote at (2) and then leaving the rest of the text unmodified.

We now change the word “probabilities” to “probability densities”, which is more accurate.

Line 172: It was initially unclear to me what you considered to be the default states, from which you later obtained the reduced (more inclusive) states. Can you clarify this? If so, please do. Note that you, on line 646, then write "the true state," which suffers the same problem. Why would this state be more "true" than the reduced set of states?

We take from this comment that our explanation of true vs reduced states was inadequate and involved inconsistent and misleading vocabulary. We have changed “the true state” to “the physical state,” and changed “the inclusive state” to “the modeled state.” We have also modified the explanation on page 10 (lines 211 through 217) to explain the distinction:

We consider the physical state to be the fully specified physical arrangement of fluorophores on the peptide molecule being analyzed. In particular, in the physical state fluorophores are unambiguously attached to specific amino acids. However, the model we used allows multiple physical states to be combined into a single modeled state. In the modeled state, the locations of the fluorophores on the peptide are not explicitly defined, and we track only the counts of each color of fluorophore and the number of amino acids on the peptide.

We have additionally made a number of related modifications to the vocabulary used in Appendix A1 - HMM state space reduction (now S1 Appendix), where appropriate.

Line 194: Why do you write "improve the theoretical complexity"? The practical complexity is also reduced by the introduced means. If you mean that the complexity order (as in the Big-O notation) is reduced, then it is better to state this as "complexity order" or similar.

Thank you, now changed to “algorithmic complexity” to be clear.

Lines 252 and 253: The "two-byte" and "eight-byte" comments are, like before, too detailed for this text. They are implementation details that can be excluded from this conceptual text.

This sentence has been removed.

Line 258: "the k nearest dye track neighbors." You need to specify the norm used to define nearest here. Even if it is just the standard Euclidean norm, please say so.

The text now mentions the use of the standard Euclidean norm.

Line 315: This line refers to the "Monte Carlo simulation section of the paper," but there are no sections named this.

Changed “Monte Carlo simulation section of the paper” to “Monte Carlo simulation Methods section.”

Line 320: I do not think the notion of "a cut-off value" is defined in this context. It becomes clear later by reading the appendices, but you should ensure the main text is understandable without the appendices or explicitly referring to them for the definition.

Added definition of a cut-off value in the kNN sub-section under methods.

Line 679: "the matrix vector" -> "The matrix-vector."

Done.

Line 708: "color of fluorophore" -> "color of the fluorophore"

Done.

Line 786: You should specify that you consider the evaluation of (31) for all t to get the T in the Big-O expressions.

Done.

Line 787: When writing O(r^2T) you should clarify that you disregard the complexity of sorting the elements (which would be on the order of SlogS where S is the number of states). I understand that your later strategy does not need to sort any entries explicitly, but this needs to be clarified at this text stage.

Thank you for finding this omission. We chose in this revision to include the time to sort in the complexity formula. We also note that the complexity of finding the r smallest of a list of S elements is given by S*log(r) if a priority queue based implementation is used. We use this result in place of the suggested S*log(S).

Line 853: Consider writing "\sigma_{kNN}" instead of "kNN \sigma" in Figure B4 to be consistent with prior notation.

Done.

Reviewer #2: Single molecule protein sequencing is a potentially revolutionary technology that could disrupt life sciences as we know it. Current methods for protein sequencing such as those based on Edman degradation rely on large ensemble measurement, whereas the authors have developed in a previous study a method whereby single molecule fluorescence detection of immobilized peptides undergoing cyclic Edman degradation could be used to identify proteins in a massively parallel format analogous to what is done with DNA on an Illumina chip. In this paper, the authors have developed a computational followup that addresses the issue of deconvoluting the noisy and complex data that arises from these fluorosequencing experiments

– a combination of multiple fluorescently labeled amino acids on a randomly cleaved peptide fragment undergoing a series of Edman degradation cycles. The authors used a hidden Markov model (HMM) to model the underlying process of degradation, fluorescence photobleaching, and random peptide detachment along with the observable – a fluorescence intensity signal at each cycle. Because the traditional HMM approach of assigning outcomes via Bayes theorem scales poorly with the vast number of possible peptide sequences and possible states, the authors developed a hybrid approach that utilizes the computationally efficient kNN classifier to reduce the complexity of the problem and apply Bayesian classification on a limited subset of possible sequences. The context of the paper, the fluorosequencing technology, is exciting and addresses a major gap in biotechnology. This paper adds a novel contribution beyond the contents of their previous work. It is also well written and technically sound. I have some minor points which I think could either be discussed and clarified in text, or if judged appropriate, be addressed experimentally to improve the manuscript.

We thank the referee for their excellent summary and assessment of the paper.

You've said that you will use the Baum Welch algorithm in the future to estimate HMM parameters – but in the meantime your model requires prior knowledge of those parameters. How much variation do you expect in the parameter values (state transition probabilities etc) and how does this propagate into classification performance? While describing your Bayesian classifier on line 137 you wrote "Transition probabilities between these states can be approximated using previously estimated success and failure rates of each step of protein fluorosequencing." Is this realistic, and if these values are assigned with 5, 10, 50% error – how will this affect the precision/recall relations downstream?

To address this point, we now include a sensitivity analysis as Supplemental Figures S9-15. In general, the algorithm is reasonably robust to variation in the parameters, with some (such as mu) more sensitive than others (such as sigma). Many parameters were more adversely affected by higher error rates in general than by differences between the model and the data (such as the dud-dye rate).

Also, for the benefit of the referee, the issue of parameter estimation is an important one and we are preparing a complete second manuscript on this topic. Relevant to this paper, however, is that the parameters we estimate from experimental datasets generally fall well within ranges swept in the new Supplemental Figures S9-15.

Along the above lines – do you know if these parameters are stable? Is it realistic that the transition probabilities and other parameters are going to be fixed and reproducible from experiment to experiment or even as time evolves within an experiment? Wondering how brittle or robust the peptide-calling is, and how much depends on accurate parameterization which, I can imagine, might be resource intensive if it must be done with every experiment.

Yes, in general we know which parameters are stable and not, since they generally arise from well-behaved physicochemical behaviors, such as photobleaching and chemical destruction of dyes (both easily measured from control experiments) and Edman rates (again measurable from

control experiments). Several rates are inferred from observation (e.g. peptide detachment rates) and less easy to isolate in control experiments. However, we have generally observed high stability in these rates unless we are specifically manipulating them in control experiments (e.g. omitting key reagents to test Edman rates, etc). We now comment on rate stability on pages 23 and 24 (lines 500 through 507) and discuss it further on pages 25 and 26 (lines 535 through 541).

On line 418 you wrote: “ It is also interesting that the HMM pruning operation is more necessary with longer peptides and more colors of fluorophores; with the trypsinized dataset labeling D/E, C, and Y, omitting pruning had little consequence, but in moving to cyanogen bromide with D/E, C, Y, and K, we observed a runtime speedup of about 1000-fold.” Could you explain the intuition behind this scaling phenomenon?

Thank you for this comment, we agree that a discussion of this scaling phenomenon would be beneficial. We’ve included the following paragraph in the text on page 24 (lines 523 through 529):

The algorithmic complexity without HMM pruning is tied to the number of states in the model, because each state must be visited at every step of the forward algorithm. The number of states in the model grows with the product of the numbers of fluorophores of each color. Pruning improves runtime by restricting to narrower ranges of fluorophore counts that the forward algorithm needs to consider at each timestep. This effect is multiplicative in the number of fluorophore colors, which we believe explains most of the improvement between these two scenarios. Longer peptides will typically have more labelable amino acids, which could amplify this effect.

We have also added a description of the computational resources used to help with interpretation of reported runtimes on page 18 (lines 382 through 386).

Reviewer #3: The paper describes a model for peptide sequence determination from the data produced from fluorosequencing - a new recently proposed technology. The authors constructed several different machine learning models, using combinations of HMM and kNN classifiers. The fluorosequencing data was simulated based on the model proposed in the previous manuscript by the same group. The authors trained and evaluated multiple prediction models using the simulated data and concluded that a hybrid HMM/kNN model achieved the balance between precision and performance. The manuscript is well-written, the computational problem is clearly explained, and the proposed models are sound. The model implementations are publicly available on github.

My only concern is the lack of testing on the real fluorosequencing data, given that the overlapping group of authors have generated such data in their previous work. Since the current

models were trained and evaluated under the same Monte-Carlo simulation model, it is unclear how the proposed models would extend to the real measurements.

We understand your concern. For the benefit of the referee, we clarify that this code has been run on real data and gives acceptable results. However, those results require too many experimental improvements to include in this paper, and without these improvements the data collected is not of sufficient quality for classification in this manner. These improvements–spanning every aspect of the sequencing process, from dye choice to dye attachment to Edman chemistry optimization to slide/surface preparation to raw signal processing–are the subject of a full second manuscript that we intend to deposit in the *bioRxiv* in the near future.

In addition, I was not able to find how the authors split their simulations into training/testing sets. Did training and testing sets contain non-overlapping peptides?

On re-reading the results section, we understand your confusion on the training/testing split. We described how the test data was generated in great detail but only briefly mentioned the training data. We have modified the document on page 18 to add greater clarity to the generation of training data for kNN. We also now clarify in the text, also on page 18, that the Bayesian HMM approach requires no explicit training data, as it is based on a direct physical model of the fluorosequencing process (lines 372 through 376).

We also added an additional clarification on pages 27 and 28 in response to your question about non-overlapping peptides in the testing and training sets (lines 591 through 596).

We have taken additional steps to address this question. Model parameters used for approximation were tuned by coarse-grained feature sweeps, and furthermore should be general enough to have no species specific effects. To validate these assumptions, we generated additional plots, similar to the plots in figure 12, for *Caenorhabditis elegans* (figure 13) and *Saccharomyces cerevisiae* (figure 14). Figure 12 was also changed to include more models.
